# Performance Analysis of a Novel Hybrid S-ALOHA/TDMA Protocol for Beta Distributed Massive MTC Access

**DOI:** 10.3390/s17122875

**Published:** 2017-12-15

**Authors:** Nannan Sui, Youyun Xu, Cong Wang, Wei Xie

**Affiliations:** 1 College of Communications Engineering, Army Engineering University of PLA, Nanjing 210007, China; nannansui@foxmail.com (N.S.); edifier@163.com (W.X.); 2 National Engineering Research Center of Communication and Network Technologies, Nanjing University of Posts and Telecommunications, Nanjing 210003, China; yyxu@vip.sina.com

**Keywords:** machine to machine (M2M), human to human (H2H), random access, hybrid MAC, dynamic pre-backoff (DPBO), equilibrium analysis

## Abstract

Simultaneous random access of massive machine type communications (MTC) devices are expected to cause congestion in the radio access network. Not only the performance of MTC, but the coexisting human to human (H2H) communications would also degrade dramatically without an appropriate medium access control (MAC) protocol. However, most existing solutions focus on the random access procedure without dealing with the sunsequent data transmission procedure. In this paper, we firstly derive a packet size threshold based on the capacity analysis of slotted ALOHA (S-ALOHA) and time division multiple access (TDMA) protocols. Then a novel hybrid S-ALOHA/TDMA MAC protocol (HSTMAC) is presented for massive MTC access, in which the resources are separated for beta distributed machine to machine (M2M) traffic with small size packets and high priority H2H traffic with large size packets. Considering access class barring (ACB) scheme as an overload control method, the system equilibrium under arbitrary retransmission limit is analyzed rigorously, which can provide insights on quality of service (QoS) guarantee. Finally, a dynamic pre-backoff (DPBO) algorithm is designed for load balance by adaptively scattering the highly synchronized M2M traffic over the transmission interval. Numerical and simulation results validate our analysis and show that the HSTMAC protocol is superior to pure S-ALOHA protocol and pure TDMA protocol. The proposed DPBO algorithm can achieve a higher success probability and resource utilization ratio with a much reduced average delay than that of uniform pre-backoff (UPBO) scheme.

## 1. Introduction

Machine type communication (MTC), also known as machine to machine (M2M) communication, is envisioned as a main enabler for the Internet of Things (IoT) [1,2]. Featuring no (or little) human intervention, M2M applications have been experiencing rapid growth in various domains, such as intelligent transport systems, smart cities, e-Health, smart grids, industry automation, monitoring and control systems, etc. [3,4]. It is expected that there will be more than 50 billion M2M devices at 2020 [5]. Unlike traditional human to human (H2H) communications, M2M traffic is characterized by massive devices, low mobility, small data transmissions and large uplink-to-downlink traffic ratio [6]. Therefore, the long term evolved (LTE) and LTE-Advanced (LTE-A) networks have to be enhanced to accommodate such differentiated M2M services and the coexisting H2H traffic [7,8,9].

**Figure 1:**
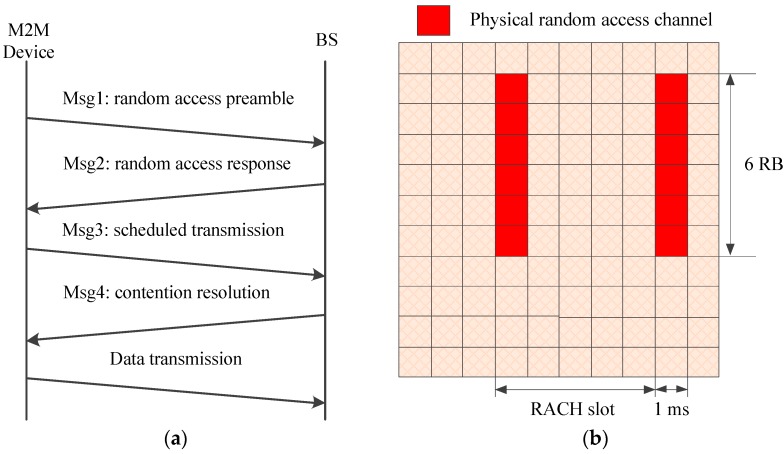
Contention-based random access procedure and physical random access channel: (**a**) Contention based random access procedure; (**b**) Physical random access channel.

A simplified model of the random access procedure in LTE/LTE-A networks is depicted in Figure 1a, which shows that four handshake messages are needed before a successful data transmission [10]. If two or more devices select the same preamble in Msg1, they will receive the same random access response (RAR) message from the base station (BS) and then send Msg3 on the same granted uplink channel. However, the BS cannot decode the superposed Msg3 accurately. Hence the colliding devices fail in this random access attempt and would send retransmission requests on the next random access channel (RACH). As a result, the main concern of most works is the RACH congestion problem caused by simultaneous random access requests from massive devices [11,12,13,14,15,16]. The existing overload control mechanisms can be classified as push-based schemes (such as access class barring (ACB), dynamic RACH resources allocation, backoff, and slotted access), pull-based scheme (group paging) and a combination of these methods.

The packet sizes of many M2M applications are usually small, some of them even on the order of bits or bytes (e.g., health or environment monitors and alarm devices) [17]. As to the random access procedure, shown in Figure 1b, just Msg1 occupies six resource blocks (RBs, a time-frequency resource unit which consists of 180 KHz in frequency domain and a time slot, i.e., 0.5 ms in time domain) in frequency and 1 ms in time (that is 12 RBs in total). Not to mention Msg3 and the retransmissions caused by collisions which can result in much more resource consumption. Thus the ratio of signaling overhead to effective payload may be unacceptable in practice [18]. However, a comprehensive investigation of both RACH congestion and the following data transmission is still lacking attention.

A few works have been aware of this problem [13,14,19,20,21]. The authors in [19] demonstrated that the supportable arrival rate of one-stage protocol, like slotted-ALOHA (S-ALOHA), is higher than that of two-stage protocol, like time division multiple access (TDMA), if the payload size is small. In [20], the LTE/LTE-A random access procedure was tailored exclusively for M2M traffic to reduce the signaling overhead. Along with the surveys of various MAC protocols for M2M communications in [13,14], we present a novel hybrid S-ALOHA and TDMA MAC protocol (HSTMAC) for M2M and H2H coexisting scenario in [21]. This paper differs from [21] in the following aspects: (i) we assume that H2H traffic follows a Poisson distribution while M2M traffic follows a Beta distribution, which represents the worst case for random access procedure; (ii) we rigorously derive the system equilibrium under any retransmission limit (*Q* ≥ 1) instead of large value assumption (*Q* ≥ 9) in [21]; (iii) based on the equilibrium analysis, a dynamic pre-backoff (DPBO) algorithm is designed for load balance of M2M traffic and quality of service (QoS) guarantee of H2H traffic. As a summary, the main contributions of this paper are as follows:

- The framework of the proposed HSTMAC protocol is detailed in this paper. The reserved uplink RBs are divided into TDMA part for H2H traffic with large size packets and S-ALOHA part for M2M traffic with small size packets. The foundation of HSTMAC protocol is proved through system capacity analysis.
- Considering the impact of ACB factor and backoff scheme, this paper derives the analytical performance of HSTMAC iteratively. Then the system equilibrium under arbitrary retransmission limit is analyzed rigorously, which can provide an insight on QoS provision.
- Based on the equilibrium analysis, a dynamic pre-backoff algorithm is designed for QoS guarantee by adaptively scattering new M2M arrivals over the transmission interval according to the highly synchronized traffic load.
- The superiority of HSTMAC in terms of success possibility, resource utilization ratio, delay and average access request number, is validated through numerical and simulation results. Compared to uniform pre-backoff (UPBO) algorithm, the proposed DPBO algorithm can achieve a higher success probability with a much reduced delay.

The remainder of this paper is organized as follows: Section 2 gives a brief review of the related works. Section 3 presents the frame structure of HSTMAC and its fundamental motivation. We exploit an iterative method to analyze the performance of HSTMAC in Section 4. Then we derive a more rigorous equilibrium analysis in Section 5, in which we also design a dynamic pre-backoff scheme. In Section 6, the performance of HSTMAC is analyzed through numerical and simulation results. We conclude this paper in Section 7.

## 2. Related Works

The differentiated characteristics of M2M traffic have been identified in [6], and are evaluated by field measurements in a large scale network in [17]. The most prominent features of M2M traffic are massive number of devices and uplink small data transmission. Therefore, the prime challenge faced with LTE/LTE-A networks is RACH congestion due to bursts of M2M access requests. Comprehensive surveys of load control mechanisms have been made in [12,13,14,15]. Third generation partnership project (3GPP) classifies these proposals into two main categories, namely push-based and pull-based methods [11]. As two of the push-based methods, ACB scheme aims at restricting the contending levels of services with different priorities, and dynamic resource allocation scheme aims at providing sufficient access opportunities according to traffic load. In ACB, BS firstly broadcasts an access barring factor *p* (0 ≤ *p* ≤ 1). Then each device generates a random number and proceeds with the following random access procedure if the number is not larger than *p*, otherwise the device has to wait for the next available access opportunity. Thus the determination of optimal ACB factor is the main concern.

In [22], both fixed and dynamic preamble allocation schemes are proposed. The ACB factor is adjusted based on load estimation without prior information. In [23], in order to maximize success probability and to satisfy the delay requirement, a Markov chain is exploited to jointly optimize the extended access barring factor and RACH resource allocation. In [24], the throughput is maximized by dynamically partitioning preambles for delay-sensitive and delay tolerant classes. In order to reduce the collision probability, the authors in [25] propose to reserve multiple uplink channels for Msg3 for a single detected preamble. 

Another solution for congestion avoidance is to reuse the available preambles and uplink resources. In [26], the devices are grouped according to their spatial locations. Then for each group, a single root can generate more preamble sequences due to the reduced radius. Moreover, if the distance between two groups is larger than a threshold, the devices from different groups are allowed to reuse the same uplink channel for Msg3 [27]. The BS can decode the overlapped information through successive interference cancelation.

As a pull-based overload control method, the performance of group paging is analyzed by an iterative method in [28]. Compared to uniform distributed traffic, the devices in group paging are usually activated simultaneously in the first access slot, which results in an extremely high system load. Due to the similar reason, surge of beta distributed M2M traffic can also cause severe performance reduction [11,29,30]. Therefore, backoff based load balance schemes for synchronized M2M traffic have been widely adopted. The authors in [31] adjust the backoff indicator dynamically to ensure the delayed devices would not enter into the next paging cycle. In [32,33], uniform pre-backoff scheme has been applied to group paging. The authors also investigate the impact of backoff window size on the system performance. Further in [34], based on the stability analysis, an optimal pre-backoff scheme has been proposed for group paging to reduce energy consumption. In [35], the UPBO mechanism is also used for beta distributed traffic.

The above works mainly focus on the M2M traffic issues. However, the H2H traffic is still taking an important role in current cellular networks. Then how to accommodate both H2H and M2M traffics at the same time becomes an urgent issue. One of the possible solutions is resources splitting. The authors in [36] analyze the impact of M2M traffic on H2H traffic. Simulation results show that H2H traffic has a better performance with RACH resource separation scheme while the M2M traffic prefers ACB scheme. A preamble set partition scheme is proposed in [37]. The impact of backoff indicator and retransmissions has been investigated by simulations. In [38,39], the RACH resources are divided into M2M section, H2H section and a sharing section. Then a mixed strategy game method in [38] and a continuous time Markov chain in [39] are used to determine the optimal allocation parameters. The authors in [40] introduce a power prioritized random access scheme. The H2H devices send preambles in a higher power level while the M2M devices in a lower power level. The BS can distinguish the requests from H2H devices from M2M devices based on two detection levels and a timing alignment matching mechanism.

Obviously, most existing works draw their attentions to RACH congestion problem without considering the following data transmission requirement. Taking signaling overhead into consideration, a theoretical analysis about supportable arrival rate against packet size is derived in [19]. The conclusion is that one stage protocol can accomodate more traffics with small size packets than two stage protocol. The advantages and drawbacks of various MAC protocols have been discussed in [13,14]. Due to the differentiated QoS requirements between M2M traffic and H2H traffic, the traditional four-way handshake random access procedure may be not applicable to such a complex scenario. Therefore, it’s a reasonable idea to design a hybrid MAC protocol to combine the strength of different MAC protocols. In [20], M2M devices can send data on the granted channels for Msg3 immediately after preamble transmissions. The authors in [41] divide the resources into contention only period and transmission only period. In contention only period, the M2M devices contend for the collision free access opportunities in the transmission only period in the way of carrier sense multiple access (CSMA) mechanism. In order to reduce the signaling overhead, the authors split preamble set into H2H part and M2M part [42]. The reserved preamble sequences for M2M devices are used in a CSMA/CA manner. However, the H2H traffic is not considered in [20,41] while the data transmission is not considered in [42]. The HSTMAC proposed in [21] divides reserved uplink resources into S-ALOHA part for M2M small data transmission and TDMA part for traditional H2H traffic. A joint dynamic ACB and resource allocation algorithm has been designed to maximize the resource utilization under a fairness constraint. In this paper, we further analyze the equilibrium performance of HSTMAC under arbitrary retransmission limit. Based on the equilibrium analysis, a dynamic pre-backoff algorithm is designed for load balance of beta distributed M2M traffic and QoS provision for H2H traffic.

## 3. The Proposed HSTMAC Framework and System Model

We first demonstrate the basis of the proposed HSTMAC through capacity analysis. Then the details of HSTMAC framework and the essential assumptions are presented.

### 3.1. Foundation of the HSTMAC Protocol

We evaluate the performance of a MAC protocol in the view of resource utilization maximization. The concept of *capacity* is defined as the maximum amount of resource blocks used for successful data transmissions. We will show how packet size affects the system capacity. 

#### 3.1.1. System Capacity of S-ALOHA Protocol

Let *d* denote the packet size and *L* denote the total available resource blocks. If the number of active devices is *X*, the average resource utilization *R* is calculated as:

(1) $$R=\delta \cdot Xe^{-\frac{X}{L/\delta }}$$

where the available data channels is *L*/*δ*. Then the well-known capacity of S-ALOHA system *C_S-ALOHA_* is obtained when *X* = *L*/*δ*:

(2) $$\begin{array}{l} C_{S-ALOHA}=Le^{-1}\\ X_{S-ALOHA}^{*}=L/\delta \end{array}$$

where $X_{S-ALOHA}^{*}$ is the maximum supportable devices of the S-ALOHA system.

#### 3.1.2. System Capacity of TDMA Protocol

The resources in TDMA system are divided into two parts for contention based random access and the following contention free data transmission. The random access procedure in TDMA can be modeled as a multi-channel S-ALOHA system [43], where each preamble is regarded as an access slot. If the RACH occupies too many RBs, the reserved data channels would be insufficient for the successful devices. On the contrary, if the reserved RBs for data channels are too many, the devices may fail due to lack of random access opportunities, which may also cause underutilization of the reserved data channels. Therefore, a tradeoff must be made for the resource allocation between random access procedure and data transmission procedure.

In order to model the uplink signaling overhead in the random access procedure, we assume that a resource block can bear *γ* random access opportunities. Let *θ* denote the proportion of RBs reserved for RACH, the resource utilization of TDMA is:

(3) $$R=\min\left\{\delta \cdot Xe^{-\frac{X}{L\theta \gamma }},L\left(1-\theta \right)\right\}$$

where *Lθγ* is the reserved random access opportunities and *L*(1−*θ*) is the reserved RBs for data channels. According to Equations (2) and (3), the resource utilization is maximized when:

(4) $$\begin{array}{l} X=L\theta \gamma \\ \delta Xe^{-\frac{X}{L\theta \gamma }}=L\left(1-\theta \right) \end{array}$$

Then the capacity of TDMA system is obtained by solving Equation (4):

(5) $$\begin{array}{l} C_{TDMA}=\delta \cdot L\theta^{*}\gamma \cdot e^{-1}=\frac{L\delta \gamma }{e+\delta \gamma }\\ \theta^{*}=\frac{1}{1+\delta \gamma /e}\\ X_{TDMA}^{*}=L\theta^{*}\gamma \end{array}$$

where $X_{TDMA}^{*}$ is the maximum supportable devices of the TDMA system.

#### 3.1.3. Packet Size Threshold

Solving the equation *C_S-ALOHA_* = *C_TDMA_*, we can get the packet size threshold:

(6) $$\bar{\delta }=\frac{1}{1-e^{-1}}\cdot \frac{1}{\gamma }$$

**Remark** **1**. *According to Equations (2) and (5), the S-ALOHA system is superior to the TDMA system if $\delta <\bar{\delta }$. On the contrary, the TDMA system is superior to the S-ALOHA system if $\delta >\bar{\delta }$ Considering that M2M traffic is usually in a small packet size while traditional H2H traffic is in a large packet size, it would be a feasible idea to design a hybrid S-ALOHA/TDMA MAC protocol for M2M and H2H coexisting scenario*.

### 3.2. Optimal ACB Parameter

For both S-ALOHA system and TDMA system, the resource utilization is a concave function with the number of active devices. In order to maximize the resource utilization, all the devices should be allowed to pass the ACB check if *X* ≤ X*, while only X* out of *X* devices can be allowed to pass the ACB check if *X* > X*. Therefore, the optimal ACB parameter for both S-ALOHA and TDMA systems is:

(7) $$p=\min\left(X^{*}/X,1\right)$$

**Remark** **2**. According to the above discussions, we can derive the concept of saturation as follows. For a certain number of available resource blocks L, the maximum number of supportable devices X* can be computed by Equations (2) and (5). We define the system is in a saturation state if the actual number of active devices is X ≥ X*, where some devices are barred to proceed the random access procedure. We define the system is in a non-saturation state if X < X*, where all the devices can participate in the contention of random access opportunities. Accordingly, the resource utilization of S-ALOHA and TDMA systems in different state can be computed as:

(8) $$R_{S-ALOHA}=\left\{ \begin{array}{lll} Le^{-1}, & if & X\geq X_{S-ALOHA}^{*}\\ \delta Xe^{-\frac{X}{L/\delta }}, & if & X<X_{S-ALOHA}^{*} \end{array}\right.$$

(9) $$\begin{array}{ll} R_{TDMA}= & \left\{ \begin{array}{lll} L(1-\theta^{*}), & if & X\geq X_{TDMA}^{*}\\ \delta Xe^{-\frac{X}{L\theta \gamma }}, & if & X<X_{TDMA}^{*} \end{array}\right.\\ s.t. & \delta Xe^{-\frac{X}{L\theta \gamma }}=L(1-\theta ) \end{array}$$

### 3.3. Frame Structure of the Proposed HSTMAC Protocol

**Figure 2:**
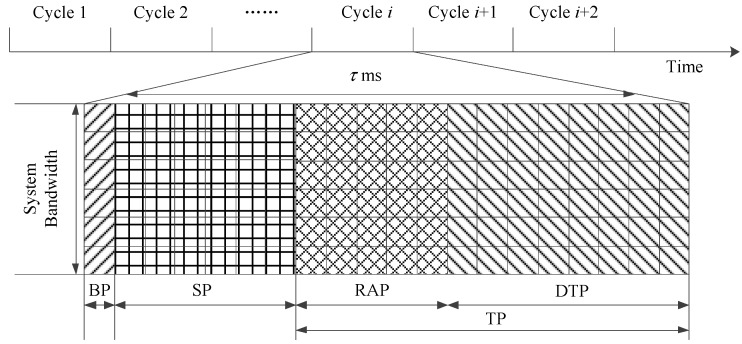
Frame structure of the proposed hybrid S-ALOHA/TDMA MAC protocol.

As shown in Figure 2, in HSTMAC the BS schedules resources on a cycle-by-cycle basis in time domain and each access cycle spans τ ms.

In order to utilize the resources more efficiently, the HSTMAC protocol mainly divides the reserved resources into three sections: broadcast period (BP) for essential downlink control information, S-ALOHA period (SP) for M2M traffic with small size packets and TDMA period (TP) for H2H traffic with large size packets. The TDMA period is further split into random access period (RAP) for contention based random access and data transmission period (DTP) for contention free packet transmission. The optimal tradeoff between RAP and DTP is made according to Equation (4). 

In the broadcast period of each cycle, BS estimates the number of active devices based on the obtained statistics and calculates the optimal resource allocation and ACB parameters for M2M and H2H traffics respectively. Then the BS broadcasts a notification message which contains these parameters to all the devices for synchronization. In this paper, we mainly focus on the uplink transmission in a single cellular cell. Let *L* denote the reserved uplink resource blocks in each cycle and let *L_SP,i_*, *L_TP,i_*, *L_RAP,i_*, *L_DTP,i_* denote the allocated RBs for the corresponding part in the *i-*th cycle, we can have:

(10) $$\begin{array}{ll} L & =L_{SP,i}+L_{TP,i}\\ & =L_{SP,i}+L_{RAP,i}+L_{DTP,i} \end{array}$$

In order to make the analysis more tractable, we assume that all the active devices, new and backlog, arrive at the beginning of a cycle and complete the transmission procedure in current cycle (no matter success or fail). Packet loss on the wireless channel is not considered in this paper yet. Hence, a packet transmission request may fail due to ACB check failure, contention collision, or insufficient data channels. The failed device would select a random backoff interval between 0 and *W_BO_* and start a retransmission attempt after the backoff counter expires. A packet will be dropped if it has not been sent successfully within the maximum *Q* transmission attempts.

In order to evaluate the performance of HSTMAC protocol in an extreme scenario, we assume that the arrival rate of H2H packets follows a Poisson distribution with mean *λ* in each access cycle and the arrival rate of M2M devices follows a Beta distribution *g*(*t*) defined in [11]:

(11) $$g(t)=\frac{t^{\alpha -1}{\left(T-t\right)}^{\beta -1}}{T^{\alpha +\beta -1}B(\alpha ,\beta )},\;t\in [0,T]$$

where *B*(*α,β*) is the Beta function and *T* is the activation time interval of M2M devices. The packet sizes of H2H traffic and M2M traffic are *δ*_1_, *δ*_2_ respectively. Let *N^M^*^2*M*^ denote the total number of M2M devices, then the number of new arrivals in cycle *i* is:

(12) $$N_{i}^{M2M}=\int_{t_{i}}^{t_{i+1}}N^{M2M}\frac{t^{\alpha -1}{\left(T-t\right)}^{\beta -1}}{T^{\alpha +\beta -1}B(\alpha ,\beta )}dt,\;t\in [0,T],i\in [1,\left\lceil \frac{T}{\tau }\right\rceil ]$$

where *t_i_* is the time of the *i*-th access cycle.

## 4. A General Model for Analytical Performance Analysis

### 4.1. Analytical Performance Analysis

Due to the beta distribution of M2M traffic, the total transmission time interval is:

(13) $$I_{\max}=\left\lceil \frac{T}{\tau }\right\rceil +\left(Q-1\right)\left(W_{BO}+1\right)$$

For the readability of this paper, we use superscripts (·)*^M^*^2*M*^ and (·)*^H^*^2*H*^ to denote the variables for M2M traffic and H2H traffic respectively. As a general analytical model used in this paper, we usually omit the superscripts for brevity without causing ambiguity. Let *X_i,j_*[*q*] denote the average number of devices which first arrive at the *j-*th cycle and perform their *q-*th transmission attempts in the *i-*th cycle. Then the new arrivals in each cycle are:

(14) $$\begin{array}{ll} X_{i,j}^{M2M}[1]= & \left\{ \begin{array}{ll} N_{i}^{M2M}, & i=j,i\leq \left\lceil \frac{T}{\tau }\right\rceil \\ 0, & otherwise \end{array}\right.\\ X_{i,j}^{H2H}[1]= & \left\{ \begin{array}{ll} \lambda , & i=j\\ 0, & i\neq j \end{array}\right. \end{array}$$

Let *X_ij_*[*q*] denote the average number of devices which perform their *q-*th transmission attempts in the *i-*th cycle and let *X_i_* denote the total number of devices which perform their transmission attempts in the *i-*th cycle. Considering the backoff indicator, we can derive that:

(15) $$X_{i}^{}[q]=\sum_{j=\max\left(i-(q-1)(W_{BO}+1),1\right)}^{i-(q-1)}X_{i,j}^{}[q]$$

(16) $$X_{i}^{}=\sum_{q=1}^{Q}X_{i}^{}[q]$$

According to the discussions in Section 3, optimal ACB parameters should be adjusted according to the actual number of devices in each cycle. For M2M traffic, the number of reserved data channels in the *i-*th cycle is:

(17) $$M_{i}^{M2M}=L_{SP,i}/\delta_{2}$$

As to the number of reserved random access opportunities for H2H traffic, we should first estimate whether the system is in a saturation state or not. According to Equations (4) and (5) and Remark 2, we can have:

(18) $$\begin{array}{ll} M_{i}^{H2H}= & \left\{ \begin{array}{ll} L_{TP,i}\theta^{*}\gamma , & X_{i}^{H2H}\geq L_{TP,i}\theta^{*}\gamma \\ L_{TP,i}\theta \gamma , & X_{i}^{H2H}<L_{TP,i}\theta^{*}\gamma \end{array}\right.\\ s.t. & \delta_{1}X_{i}^{H2H}e^{-\frac{X_{i}^{H2H}}{L_{TP,i}\theta \gamma }}=L_{TP,i}(1-\theta ) \end{array}$$

Therefore, the optimal ACB parameter can be computed as:

(19) $$p_{i}=\min\left(M_{i}^{}/X_{i}^{},1\right)$$

So the average number of devices which pass the ACB check is *X_i_p_i_* and the average number of successful devices is:

(20) $$Z_{i}^{}=X_{i}^{}p_{i}e^{-\frac{X_{i}p_{i}}{M_{i}}}$$

Because each device has the same success probability, if we let *Z_i,j_*[*q*] denote the average number of devices which first arrive at the *j-*th cycle and succeed in their *q-*th transmission attempts in the *i-*th cycle, we can derive that:

(21) $$Z_{i,j}^{}[q]=\frac{X_{i,j}^{}[q]}{X_{i}^{}}Z_{i}^{}$$

(22) $$\begin{array}{ll} Z_{i} & =\sum_{q=1}^{Q}Z_{i}^{}[q]\\ & =\sum_{q=1}^{Q}\sum_{j=\max\left(i-(q-1)(W_{BO}+1),1\right)}^{i-(q-1)}Z_{i,j}^{}[q] \end{array}$$

where *Z_i_*[*q*] is the number of devices which succeed in their *q-*th access attempts in the *i-*th cycle.

In each cycle, the failed device which uses up its retransmission opportunities would drop the packet and experiences a final transmission failure. Otherwise, the failed device would randomly select a backoff interval *ω_BO_* ∈ [0,*W_BO_*] and retry access to the network in the *i*+*ω_BO_*+1-th cycle. So the backlogged devices due to backoff can be computed as follows:

(23) $$X_{i,j}^{}[q]=\frac{1}{W_{BO}+1}\sum_{k=\max\left(i-(W_{BO}+1),1\right)}^{i-1}\left(X_{k,j}^{}[q-1]-Z_{k,j}^{}[q-1]\right),2\leq i\leq I_{\max},2\leq q\leq Q$$

(24) $$\begin{array}{ll} X_{i}^{}[q] & =\sum_{j=\max\left[i-(q-1)(W_{BO}+1),1\right]}^{i-(q-1)}X_{i,j}^{}[q]\\ & =\sum_{j=\max\left[i-(q-1)(W_{BO}+1),1\right]}^{i-(q-1)}\frac{1}{W_{BO}+1}\sum_{k=\max\left(i-(W_{BO}+1),1\right)}^{i-1}\left(X_{k,j}^{}[q-1]-Z_{k,j}^{}[q-1]\right)\\ & =\frac{1}{W_{BO}+1}\sum_{k=\max\left(i-(W_{BO}+1),1\right)}^{i-1}\sum_{j=\max\left[i-(q-1)(W_{BO}+1),1\right]}^{i-(q-1)}\left(X_{k,j}^{}[q-1]-Z_{k,j}^{}[q-1]\right)\\ & =\frac{1}{W_{BO}+1}\sum_{k=\max\left(i-(W_{BO}+1),1\right)}^{i-1}\left(X_{k}^{}[q-1]-Z_{k}^{}[q-1]\right) \end{array}$$

### 4.2. Performance Metrics

Four performance metrics, which are average success probability, resource utilization ratio, the statistics of delay and the number of transmission request, are chosen to evaluate the performance of the HSTMAC protocol. These four performance metrics are defined as follows.

The average success probability, i.e., *P_S_*, is defined as the ratio of the total number of successful devices to the total number of newly arrived devices during *I*_max_ cycles:

(25) $$\begin{array}{ll} P_{S}^{H2H} & =\frac{\sum_{i=1}^{I_{\max}}Z_{i}^{H2H}}{\lambda I_{\max}}\\ & =\frac{\sum_{i=1}^{I_{\max}}\sum_{q=1}^{Q}Z_{i}^{H2H}[q]}{\lambda I_{\max}} \end{array}$$

(26) $$\begin{array}{ll} P_{S}^{M2M} & =\frac{\sum_{i=1}^{I_{\max}}Z_{i}^{M2M}}{N^{M2M}}\\ & =\frac{\sum_{i=1}^{I_{\max}}\sum_{q=1}^{Q}Z_{i}^{M2M}[q]}{N^{M2M}} \end{array}$$

The resource utilization ratio *U* is defined as the ratio of the total number of RBs used for successful packet transmissions to the total number of reserved RBs:

(27) $$U^{H2H}=\frac{\sum_{i=1}^{I_{\max}}\sum_{q=1}^{Q}\delta_{1}Z_{i}^{H2H}[q]}{\sum_{i=1}^{I_{\max}}L_{TP,i}}$$

(28) $$U^{M2M}=\frac{\sum_{i=1}^{I_{\max}}\sum_{q=1}^{Q}\delta_{2}Z_{i}^{M2M}[q]}{\sum_{i=1}^{I_{\max}}L_{SP,i}}$$

The delay of a successful device is defined as the time intervals (granularity in cycle) between its first arrival time and the time when it sends its packet successfully. So the statistical average delay *D* is computed as:

(29) $$D=\frac{\sum_{i=1}^{I_{\max}}\sum_{q=1}^{Q}\sum_{j=\max\left[i-(q-1)(W_{BO}+1),1\right]}^{i-(q-1)}\left(i-j)Z_{i,j}^{}[q\right]}{\sum_{i=1}^{I_{\max}}\sum_{q=1}^{Q}Z_{i}^{}[q]}$$

The average request number *B* is defined as the average number of access attempts that a successful device has conducted:

(30) $$B=\frac{\sum_{i=1}^{I_{\max}}\sum_{q=1}^{Q}qZ_{i}^{}[q]}{\sum_{i=1}^{I_{\max}}\sum_{q=1}^{Q}Z_{i}^{}[q]}$$

## 5. System Equilibrium Analysis under Arbitrary Transmission Limit

### 5.1. System Balance Equation

The authors in [44] analyzed the system equilibrium point of the random access procedure without considering the following data transmission procedure and ACB scheme. In [21], the system equilibrium is derived asymptotically for joint optimization of random access and data transmission under a large transmission limit (*Q* ≥ 9). Compared to [21], this section would provide a more rigorous system equilibrium analysis under arbitrary transmission limit *Q*(*Q* ≥ 1).

Apparently, when the system with uniform distributed traffic converges to the equilibrium state, there must be *X_i_* = *X_j_*, ∀*i,j* and *Z_i_* = *Z_j_*, ∀*i,j*. Let *f* denote the one-shot success probability in each cycle, according to Equation (24) we can derive that:

(31) $$\begin{array}{ll} X_{i}^{}[2] & =\frac{1}{W_{BO}+1}\sum_{k=i-(W_{BO}+1)}^{i-1}\left(X_{k}^{}[1]-Z_{k}^{}[1]\right)\\ & =\frac{1}{W_{BO}+1}(W_{BO}+1)(X_{i-1}^{}[1]-Z_{i-1}^{}[1])\\ & =X_{i-1}^{}[1]-Z_{i-1}^{}[1]\\ & =\lambda (1-f) \end{array}$$

where *λ* is the average number of new arrivals. Therefore, in a recursive way, we can have the following equation:

(32) $$\begin{array}{lll} X_{i}^{}[q] & =X_{i-1}^{}[q-1]-Z_{i-1}^{}[q-1] & \\ & =\lambda {\left(1-f\right)}^{q-1}, & 2\leq q\leq Q \end{array}$$

Omitting the time subscript *i*, the system balance equation is derived as follows:

(33) $$\begin{array}{ll} X & =\sum_{q=1}^{Q}X[q]\\ & =\sum_{q=1}^{Q}\lambda {\left(1-f\right)}^{q-1}\\ & =\lambda \frac{1-{\left(1-f\right)}^{Q}}{f} \end{array}$$

**Remark** **3.** *According to Equations (31)–(33), we can find that the components of devices in each cycle are the same with and without backoff scheme. Therefore, the uniform backoff scheme has no impact on the system performance except delay. Then according to the definitions in Subsection 4.2, the performance metrics in equilibrium state are computed as:*

(34) $$\begin{array}{ll} P_{S} & =1-{\left(1-f\right)}^{Q}\\ U & =\frac{\delta \sum_{q=1}^{Q}\lambda {\left(1-f\right)}^{q-1}f}{L}\\ & =\frac{\delta \lambda P_{S}}{L}\\ B & =\sum_{q=1}^{Q}q\cdot \frac{{\left(1-f\right)}^{q-1}f}{1-{\left(1-f\right)}^{Q}}\\ D & =\sum_{q=1}^{Q}(q-1)\frac{1+\left(W_{BO}+1\right)}{2}\cdot \frac{{\left(1-f\right)}^{q-1}f}{1-{\left(1-f\right)}^{Q}} \end{array}$$

where *L* is the reserved resource blocks and $\frac{1+\left(W_{BO}+1\right)}{2}$ is the average delay caused by backoff.

### 5.2. Equilibrium Analysis for S-ALOHA System

We define the system load as $\rho =\frac{\lambda }{L/\delta }$. When ACB is enabled, the average number of contending devices on a data channel is defined as $s=\frac{Xp}{L/\delta }$. Let p=1, i.e., disable the ACB scheme, we can have the same system balance equation as Equation (11) in [44]:

(35) $$\begin{array}{ll} f & =e^{-\frac{X}{L/\delta }}=e^{-s}\\ \frac{X}{\lambda } & =\frac{s}{\rho }=\frac{1-{\left(1-e^{-s}\right)}^{Q}}{e^{-s}} \end{array}$$

That is to say if the ACB is not considered, we can obtain all the system equilibrium points by solving Equation (35). Then according to [44], there may be multiple solutions and some of which are on *s* ∈ (1,+∞). However, when ACB scheme is enabled, there must be $s=\frac{Xp}{L/\delta }\leq 1$. Therefore, we have to discuss the system equilibrium under different system load and retransmission limit case by case.

#### 5.2.1. Equilibrium Analysis in the Saturation State

If the S-ALOHA system is in a saturation state when it converges to the equilibrium state, there must be *s* = 1 according to Section 3.2. A successful device should first pass the ACB check and then succeed in the data transmission procedure without collision. Therefore, we can rewrite the system balance equation as follows:

(36) $$\begin{array}{ll} f & =pe^{-\frac{Xp}{L/\delta }}\\ & =pe^{-1}\\ X & =\frac{L/\delta }{p}\\ & =\lambda \frac{1-{\left(1-f\right)}^{Q}}{f} \end{array}$$

Then we can derive that:

(37) $$\begin{array}{l} p=e\left[1-{\left(1-\frac{L/\delta }{e\lambda }\right)}^{1/Q}\right]\\ f=1-{\left(1-\frac{L/\delta }{e\lambda }\right)}^{1/Q}\\ P_{S}=\frac{L/\delta }{e\lambda } \end{array}$$

Due to ACB scheme, there must have 0 ≤ *p* ≤ 1, so we can derive the following inequation:

(38) $$0\leq L\leq e\lambda \delta \left[1-{\left(1-e^{-1}\right)}^{Q}\right]$$

In this paper, we define the saturation threshold in the equilibrium state as:

(39) $$L_{th}^{S-ALOHA}=e\lambda \delta \left[1-{\left(1-e^{-1}\right)}^{Q}\right]$$

When $L=\;L_{th}^{S-ALOHA}$, the system load *ρ_th_* at the saturation threshold is obtained as:

(40) $$\rho_{th}^{}=\frac{\lambda }{L_{th}^{S-ALOHA}/\delta }=\frac{e^{-1}}{\left[1-{\left(1-e^{-1}\right)}^{Q}\right]}$$

**Remark** **4.** *Based on the above analysis, there must be $\rho \geq \rho_{th}\left(L\leq \;L_{th}^{S-ALOHA}\right)$ if the system converges to a saturated equilibrium state. Until this step we just have demonstrated that $\rho \geq \rho_{th}\left(L\leq \;L_{th}^{S-ALOHA}\right)$ is the necessary condition for a saturated equilibrium state. In the following subsections, we still need to prove that $\rho \geq \rho_{th}\left(L\leq \;L_{th}^{S-ALOHA}\right)$ is also the sufficient condition to ensure the system is saturated and to prove that there is only one equilibrium point on s∈ (0,1] if ρ < ρ_th_*.

#### 5.2.2. Equilibrium Analysis in the Non-Saturation State for *Q* ≤ 8

Let we define function *h*(*s*) as follows:

(41) $$h(s):=\frac{1-{\left(1-e^{-s}\right)}^{Q}}{e^{-s}}$$

**Figure 3:**
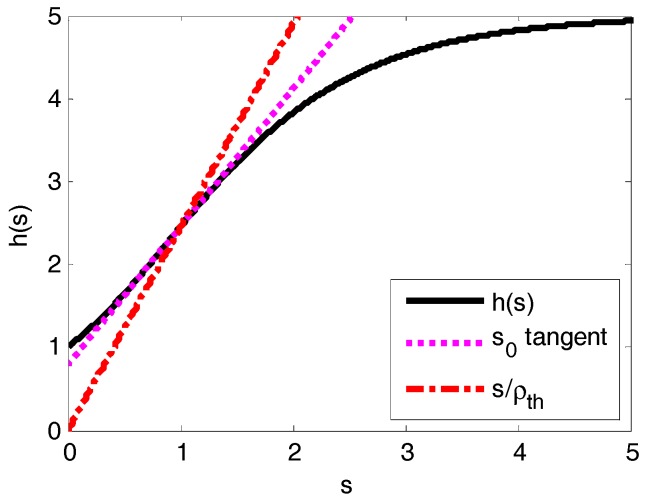
Function *h*(*s*) for *Q* = 5, *s*_0_ is the inflexion point of *h*(*s*).

Obviously, the solutions for Equation (35) are the cross points of line s/*ρ* and curve *h*(*s*). Function *h*(*s*) has exactly one convex piece on (0,*s*_0_) and one concave piece on (*s*_0_,+∞) for *Q ≥* 1. Especially for *Q* ≤ 8, there is only a single solution for Equation (35) on (0,+∞) (the proof is not a contribution of this paper, readers may refer to [44] for more details). Apparently, s/*ρ_th_* has a cross point with *h*(*s*) at point *s* = 1. Therefore, as shown in Figure 3, there must be precisely a single equilibrium point on (0,1) if *ρ* < *ρ_th_*. On the other hand, there must be precisely a single solution for Equation (35) on (1,+∞) if *ρ* > *ρ_th_*, which means that the system is in a saturation state.

#### 5.2.3. Equilibrium Analysis in the Non-Saturation State for *Q* ≥ 9

**Figure 4:**
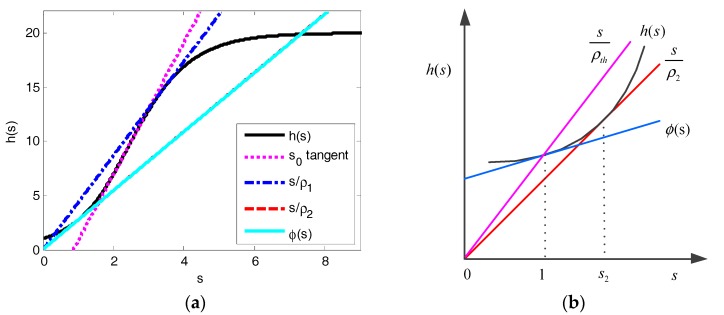
Function *h*(*s*) for *Q* = 20, *s*_0_ is the inflexion point of *h*(*s*): (**a**) Function *h*(*s*); (**b**) Illustration for proof of *s*_2_ ≥ 1.

For *Q* ≥ 9, there are two load boundaries 0 < *ρ*_1_ < *ρ*_2_ < ∞. Actually, as shown in Figure 4a, *s*/*ρ*_1_ is the tangent of *h*(*s*) on (*s*_0_,+∞) and *s*/*ρ*_2_ is the tangent of *h*(*s*) on (0,*s*_0_). Let *s*_2_ denote the point of tangency of *s*/*ρ*_2_ and *h*(*s*), we can derive that:

(i) for *ρ* > *ρ*_2_, there is only a single equilibrium point on (*s*_2_,+∞);
(ii) for *ρ* < *ρ*_2_, there is only a single equilibrium point on (0,*s*_2_) and there are two equilibrium points on (*s*_2_,+∞) for *ρ*_1_ > *ρ* > *ρ*_2_ and one equilibrium point on (*s*_2_,+∞) for *ρ* > *ρ*_1_.

As shown in Figure 4b, if we can prove that *s*_2_ ≥ 1, there must be precisely a single equilibrium point on (0,1) for *ρ* < *ρ_th_*.

As shown in Figure 4b, we can have the tangent *φ*(*s*) of *h*(*s*) at *s* = 1:

(42) $$\phi (s)=h^{\prime }(1)s-h^{\prime }(1)+h(1)$$

The vertical intercept of *φ*(*s*) is computed as:

(43) $$\begin{array}{ll} \phi (0) & =-h^{\prime }(1)+h(1)\\ & =Q{\left(1-e^{-1}\right)}^{Q-1}\\ & >0 \end{array}$$

So we must have *s*_2_ ≥ 1. That is to say, there is only a single equilibrium point on (0,1) if *ρ* < *ρ_th_*. For *ρ* > *ρ_th_*, all the solutions for Equation (35) are on (1,+∞), which means that the system is in a saturation state.

To sum up, if the ACB scheme is enabled, we can derive the following conclusions for S-ALOHA system under *Q* ≥ 1

1. If $L\leq \;L_{th}^{S-ALOHA}$, i.e., *ρ* ≥ *ρ_th_*, the system would surely converge to a saturated equilibrium state. Therefore, the equilibrium point cannot be computed by Equation (35) directly and the ACB scheme would ensure that *s* = 1. Then the average success probability and resource utilization ratio can be computed as:
  (44) $$\begin{array}{ll} P_{S} & =\frac{L/\delta }{e\lambda }\\ U & =\frac{\delta \lambda P_{S}}{L}=e^{-1} \end{array}$$
2. If $\;L>\;L_{th}^{S-ALOHA}$, i.e., *ρ* < *ρ_th_*, the ACB scheme would ensure the system converges to an equilibrium state which is non-saturate. The single unique equilibrium point *s* ∈ (0,1) can be computed by Equation (35). In the non-saturation state we can have:
  (45) $$\begin{array}{ll} P_{S} & =1-{\left(1-e^{-s}\right)}^{Q}\\ U & =\frac{\delta \lambda P_{S}}{L} \end{array}$$

### 5.3. Equilibrium Analysis for TDMA System

For the TDMA system, we can define the system load as *ρ* = *λ*/*Lθγ*. Considering the ACB factor, the average number of contending devices on a random access opportunity is defined as *s* = *Xp*/*Lθγ*. Substituting *ρ*,*s* into Equation (35), we can have the balance equation for the TDMA system. The following condition must be satisfied:

(46) $$\begin{array}{ll} \delta Xpe^{-\frac{Xp}{L\theta \gamma }} & =\delta Xpe^{-s}\\ & =L\left(1-\theta \right) \end{array}$$

The capacity of TDMA system is achieved when *θ* = θ*. Hence, in a similar way to the S-ALOHA system, we can obtain the saturation threshold in the equilibrium state as:

(47) $$L_{th}^{TDMA}=\frac{e\lambda \left[1-{\left(1-e^{-1}\right)}^{Q}\right]}{\theta^{*}\gamma }$$

Therefore, we can derive the following conclusions for TDMA system under *Q* ≥ 1:

1. If $L<\;L_{th}^{TDMA}$, the system would surely converge to a saturated equilibrium state. Therefore, the ACB scheme would ensure that *s* = 1. Then the average success probability and resource utilization ratio can be computed as:
  (48) $$\begin{array}{cc} p & =e\left[1-{\left(1-\frac{L\theta^{*}\gamma }{e\lambda }\right)}^{1/Q}\right]\\ f & =1-{\left(1-\frac{L\theta^{*}\gamma }{e\lambda }\right)}^{1/Q}\\ P_{S} & =\frac{L\theta^{*}\gamma }{e\lambda }\\ U & =\frac{\delta \lambda P_{S}}{L}=1-\theta^{*} \end{array}$$
2. If $L>\;L_{th}^{TDMA}$, the ACB scheme would ensure the system converges to an equilibrium state which is non-saturate. The single unique equilibrium point *s* ∈ (0,1) and the corresponding *θ* can be computed by Equations (35) and (46). In the non-saturation state we can have:
  (49) $$\begin{array}{ll} P_{S} & =1-{\left(1-e^{-s}\right)}^{Q}\\ U & =\frac{\delta \lambda P_{S}}{L}=1-\theta \end{array}$$

### 5.4. QoS Guaranteed Resource Allocation and Dynamic Pre-Backoff Algorithm

For Poisson distributed H2H traffic, a backoff scheme can only increase delays without improving the other performance metrics. Hence, in this paper the backoff scheme is not used for H2H traffic. We will first satisfy the resource requirement of H2H traffic due to its high priority. Then according to the prior equilibrium analysis, the reserved resource blocks *L_TP_* for a given QoS requirement can be computed by Equations (34), (48) and (49).

As to the M2M traffic, the maximum supportable arrival rate for a given available resources *L_SP_* can be computed by Equation (39):

(50) $$\lambda_{th}^{M2M}=\frac{L_{SP}}{\delta_{2}e\left[1-{\left(1-e^{-1}\right)}^{Q}\right]}$$

The pre-backoff scheme should be enabled to balance the system load for beta distributed M2M traffic if $\lambda \geq \lambda_{th}^{M2M}$. Therefore in this paper, we design an aggressively increase conservatively decrease based dynamic pre-backoff scheme as stated in Algorithm 1, in which *η* is a pre-defined time parameter to control the pre-backoff window size. In the view of the base station, *X_i_*[1] is the actual rate of new arrivals. Each new arrival in the *i-*th cycle would randomly select a backoff interval *ω_PBO_*∈ [0,*W_PBO_*] and send its first access request in the *i* + *ω_PBO_*-th cycle.

**Algorithm** **1:** Dynamic pre-backoff algorithm (DPBO) **Input**: $L,\lambda ,N_{M2M},T,\alpha ,\beta ,\theta ,\gamma ,\tau ,\delta_{1},\delta_{2},\eta ,Q,W_{BO}$, QoS requirements of H2H traffic **Initialization:**  Solve Equation (13) and return $I_{\max}$;  $X_{i}^{M2M}[1]=0,\;\forall i\in [1,I_{\max}]$;  $W_{PBO}=0$; **Step1:** Solve Equations (34), (48) and (49) and return $L_{TP}$;    Solve Equation (10) and return $L_{SP}$;    Solve Equation (50) and return $\lambda_{th}^{M2M}$; **Step2: for** each cycle $i=1:I_{\max}$ **do**     Solve Equation (12) and return $N_{i}^{M2M}$;     # Newly arrived M2M devices execute pre-backoff procedure      **for** $k=1:W_{PBO}+1$ **do**      $X_{i+k-1}^{M2M}[1]=X_{i+k-1}^{M2M}[1]+\frac{1}{W_{PBO}+1}N_{i}^{M2M}$;      **end for**     # Adjust the pre-backoff window size dynamically      **if** $X_{i}^{M2M}[1]\geq \lambda_{th}^{M2M}$       $W_{PBO}=W_{PBO}+1$;       **else if** $X_{i}^{M2M}[1]<\lambda_{th}^{M2M}$ continuously in time interval [i−η,i]         $W_{PBO}=W_{PBO}-1$;      **end if**      $I_{\max}=\max\left[i+W_{PBO}+\left(Q-1\right)\left(W_{BO}+1\right),I_{\max}\right]$    **end for**

## 6. Numerical and Simulation Results

In this paper, we consider a TDD cellular cell and the corresponding system parameters are defined in Table 1 [11]. The simulations are performed using Matlab and the Monte-Carlo results are obtained after 1000 runs. As to the QoS provision for H2H traffic, we assume that the reserved RBs would ensure H2H traffic converges to the equilibrium state which is precisely at the saturation threshold. The corresponding success probability at the saturation threshold under different transmission limit is listed in Table 2. In order to compare the performance of the proposed HSTMAC protocol with pure S-ALOHA protocol and pure TDMA protocol, we assume that the resources in S-ALOHA and TDMA protocols are also split for M2M traffic and H2H traffic separately. 

### 6.1. System Performance versus Packet Size

In this and the next subsections, we will validate the analysis of system capacity and system equilibrium for the pure S-ALOHA protocol and pure TDMA protocol. For this purpose, we assume that the arrival rate of new packets follows a Poisson distribution and the evaluation time is set to 500 cycles.

**Figure 5:**
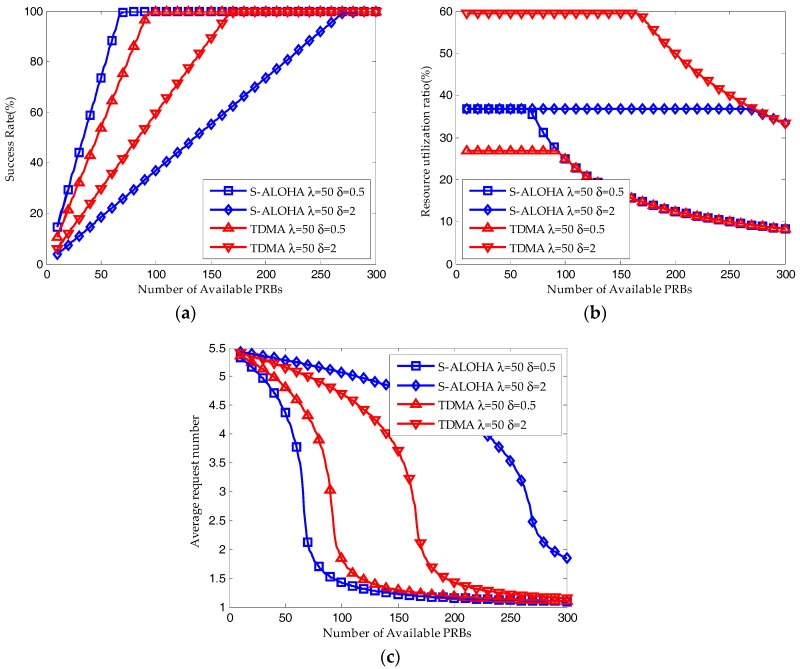
Performance results of pure S-ALOHA protocol and pure TDMA protocol with different packet size (Q = 10, W_BO_ = 0): (**a**) Average success probability; (**b**) Resource utilization ratio; (**c**) Average request number.

Shown in Figure 5, the system performance of S-ALOHA and TDMA protocols with different packet size are derived by the iterative method. For small packet size *δ =* 0.5, as shown in Figure 5a,b, when the available resource blocks are insufficient, both the average success probability and resource utilization ratio of S-ALOHA protocol is higher than that of TDMA protocol. At the same time, as shown in Figure 5c, the average request number of S-ALOHA protocol is lower than that of TDMA protocol. When the number of available resource blocks is large enough, the success probability and resource utilization ratio of S-ALOHA protocol are almost the same with that of TDMA protocol while the average request number of S-ALOHA protocol is still lower than that of TDMA protocol. For large packet size *δ =* 2, on the contrary, when the available resource blocks are insufficient, both the average success probability and resource utilization ratio of S-ALOHA protocol is lower than that of TDMA protocol and the average request number of S-ALOHA protocol is higher than that of TDMA protocol. When the number of available resource blocks is large enough, the success probability and resource utilization ratio of S-ALOHA protocol are almost the same with that of TDMA protocol while the average request number of S-ALOHA protocol is still higher than that of TDMA protocol. Therefore, we can validate the analysis in Section 3.1 and make a conclusion that the S-ALOHA protocol is superior to the TDMA protocol for small packet transmission while the TDMA protocol is superior to the S-ALOHA protocol for large packet transmission.

### 6.2. Equlibrium Analysis

**Figure 6:**
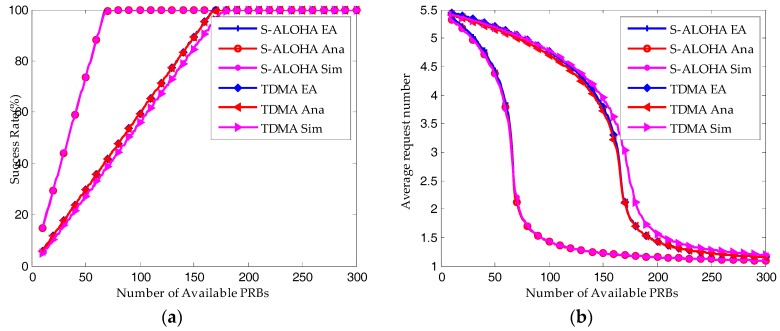
Comparison of simulation results with equilibrium analysis and iterative analytical results (λ = 50, Q = 10, W_BO_ = 0): (**a**) Average success probability; (**b**) Average request number.

As shown in Figure 6, the simulation results (Sim in the figure) match well with the equilibrium analysis (EA in the figure) and analytical results (Ana in the figure). For S-ALOHA protocol, the maximum difference is found in the average request number, which is 5.54%. For TDMA protocol, though the simulation results are shifting to the right, they have the same tendencies with the equilibrium analysis and analytical results.

**Figure 7:**
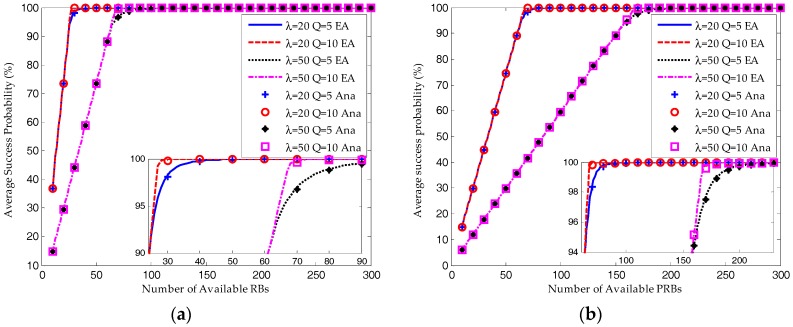
Comparison of average success probability between equilibrium analysis and iterative analytical results (W_BO_ = 0): (**a**) S-ALOHA protocol; (**b**) TDMA protocol.

**Figure 8:**
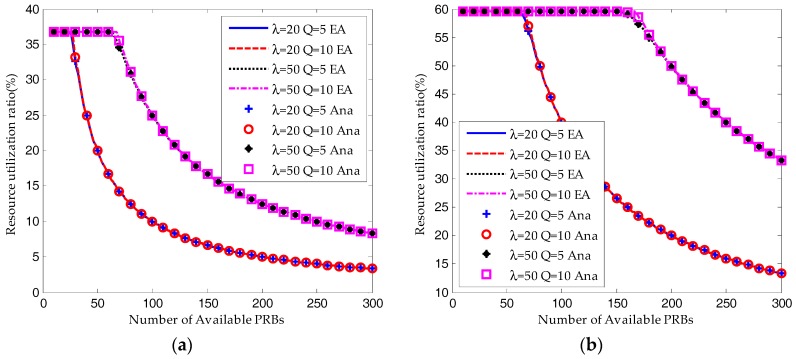
Comparison of resource utilization ratio between equilibrium analysis and iterative analytical results (W_BO_ = 0): (**a**) S-ALOHA protocol; (**b**) TDMA protocol.

As shown in Figure 7 and Figure 8, when the available RBs are insufficient, the ACB scheme would ensure the system converges to a saturated equilibrium state. Then according to Equations (44) and (48), for both the S-ALOHA and TDMA protocols, the average success probability is increasing linearly with the number of available RBs and the resource utilization ratio remains constant. The transmission limit has no impact on the success probability and resource utilization ratio under such a condition. However, as shown in Figure 8, the average request number for *Q* = 10 is much higher than that for *Q* = 5.

As shown in Figure 7, when the number of available RBs is around the saturation threshold, the average success probability is still increasing but the growth is decreasing. The success probability for *Q* = 10 is a little higher than that for *Q* = 5. And the request number for *Q* = 10 is still higher than that for *Q* = 5. This demonstrates that the device can increase its success probability by trying more times.

**Figure 9:**
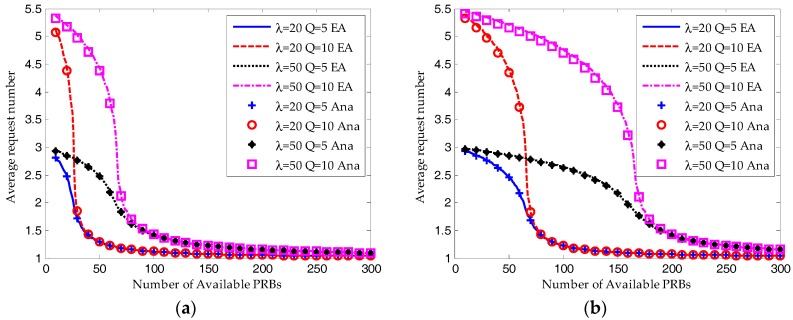
Comparison of request number between equilibrium analysis and iterative analytical results (W_BO_ = 0): (**a**) S-ALOHA protocol; (**b**) TDMA protocol.

When the number of RBs is large enough, almost all the devices can succeed in their transmission attempts. However, the resource utilization ratio is decreasing due to the underutilization of RBs. On the other hand, as shown in Figure 7, Figure 8 and Figure 9, the performance results for *Q* = 10 and *Q* = 5 are almost the same.

As a conclusion, the system performance can be improved dramatically by increasing the available resources when it is in the saturation state. However, the performance improvement would be slight when the system is in the non-saturation state. Increasing the transmission limit can impose a positive impact on the success probability at a cost of increasing in the average request number, which means more energy consumption and higher delay.

### 6.3. Performance of the HSTMAC Protocol without Backoff Scheme

**Figure 10:**
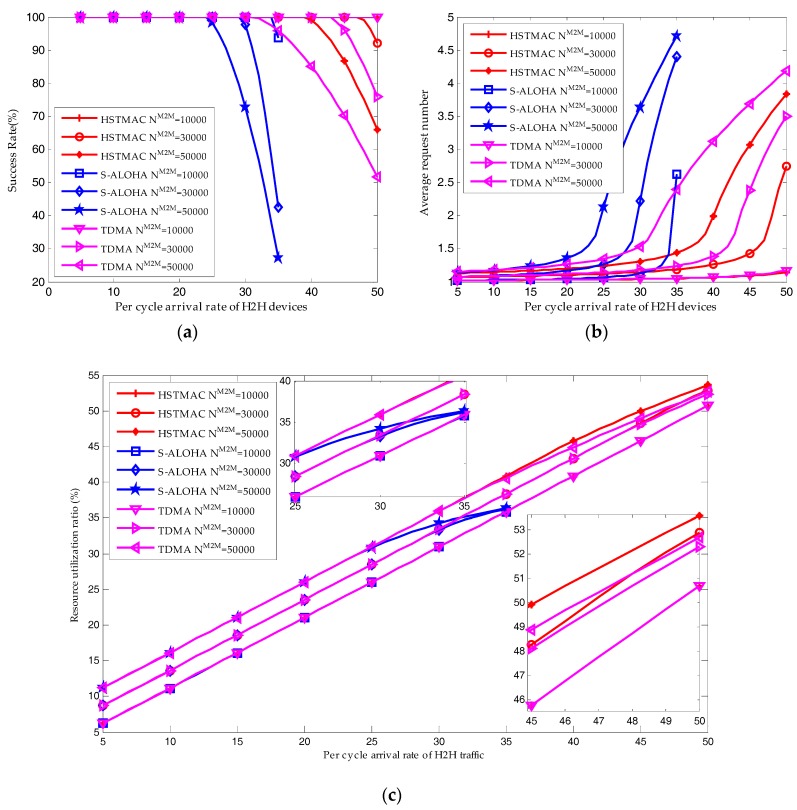
Performance comparison of the proposed HSTMAC protocol with pure S-ALOHA protocol and pure TDMA protocol (Q = 10, W_BO_ = 0): (**a**) Average success probability of M2M traffic; (**b**) Average request number of M2M traffic; (**c**) Resource utilization ratio.

As shown in Figure 10a, the average success probability of M2M traffic is always decreasing with the arrival rate of H2H packets. On the other hand, shown in Figure 10c, the average resource utilization ratio is increasing with the arrival rate of H2H packets. That is because the number of reserved RBs for M2M traffic is decreasing and the reserved resources are insufficient to satisfy the QoS requirement of M2M traffic. Under such a condition, according to the equilibrium analysis in Section 5, the resource utilization ratio is increasing with the system load.

As the number of M2M devices is increasing from 10,000 to 50,000, there is a growth in the system load, which results in a higher resource utilization ratio. The reserved RBs are insufficient to satisfy the QoS requirement of M2M traffic. Hence, as shown in Figure 10a,b, there is a remarkable decrease in the average success probability and there is a remarkable increase in the average request number.

Apparently, Figure 10 shows that the average success probability and resource utilization ratio of the proposed HSTMAC protocol are always higher than that of S-ALOHA and TDMA protocols while the average request number is always lower than that of S-ALOHA and TDMA protocols. The reason is that S-ALOHA is appropriate for small size packet transmission while TDMA is appropriate for large size packet transmission. In order to satisfy the QoS requirement of H2H traffic, the number of reserved RBs for H2H traffic in S-ALOHA protocol is much higher than that in TDMA protocol. As shown in Figure 10, for a high H2H traffic load in the S-ALOHA protocol, the QoS cannot be satisfied even if H2H traffic occupies all the RBs. Therefore, comparing the HSTMAC protocol to the TDMA protocol, the number of reserved RBs for M2M traffic are the same. However, comparing the HSTMAC protocol to the S-ALOHA protocol, the number of reserved resource blocks for M2M traffic is much higher. As a result, the proposed HSTMAC protocol is superior to the pure TDMA protocol and pure S-ALOHA protocol especially in a high system load scenario.

### 6.4. Performance of the HSTMAC Protocol with Dynamic Pre-Backoff Scheme

**Figure 11:**
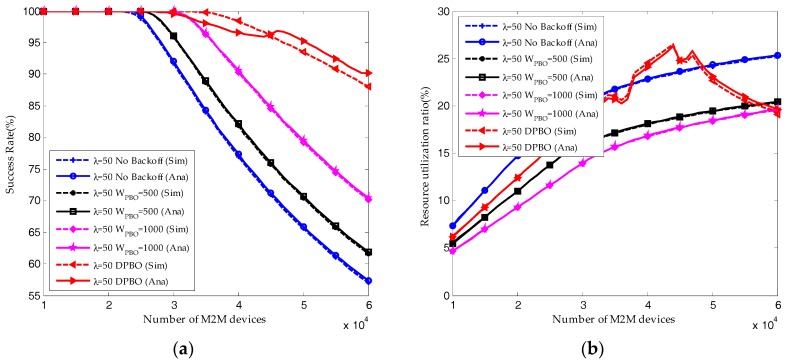
Performance comparisons of HSTMAC protocol between analytical and simulation results (λ = 50, Q = 10, W_BO_ = 20): (**a**) Average success probability of M2M traffic; (**b**) Average resource utilization ratio of M2M traffic.

As shown in Figure 11, we validate the analytical analysis of DPBO algorithm through simulation results. For DPBO algorithm, jitters can be found in the resource utilization ratio and average request number. The reason is that the pre-backoff window size of DPBO is changing dynamically according to the traffic load, which results in the dynamically changing of the transmission interval $I_{\max}$ and the distribution of devices in different transmission stages.

**Figure 12:**
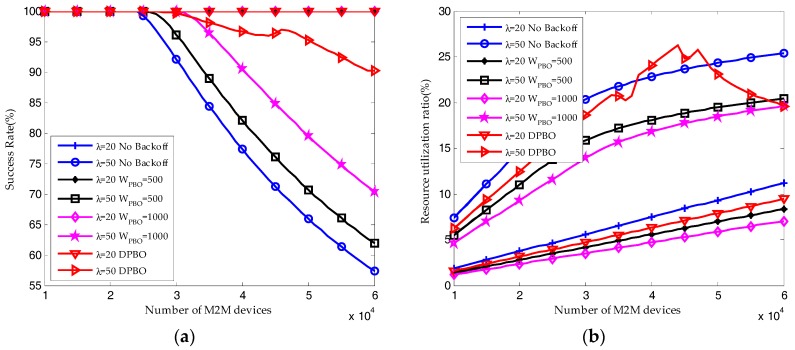
Performance results of HSTMAC protocol with dynamic pre-backoff scheme (Q = 10, W_BO_ = 20): (**a**) Average success probability of M2M traffic; (**b**) Average resource utilization ratio of M2M traffic.

When the arrival rate of H2H packets is *λ* = 20, the reserved RBs are sufficient to satisfy the QoS requirement of M2M traffic. Shown in Figure 12a, almost all the M2M devices can send their packets successfully, and the resource utilization ratio of M2M traffic shown in Figure 12b is increasing linearly with the number of M2M devices. Under this condition, the pre-backoff window size in DPBO algorithm would remain close to zero. The time interval *I*_max_ is increasing with the pre-backoff and backoff window size. Hence, as shown in Figure 10 and Figure 11, the resource utilization ratio and the average request number in the DPBO algorithm is higher while the average delay is much lower than that in the UPBO algorithm.

**Figure 13:**
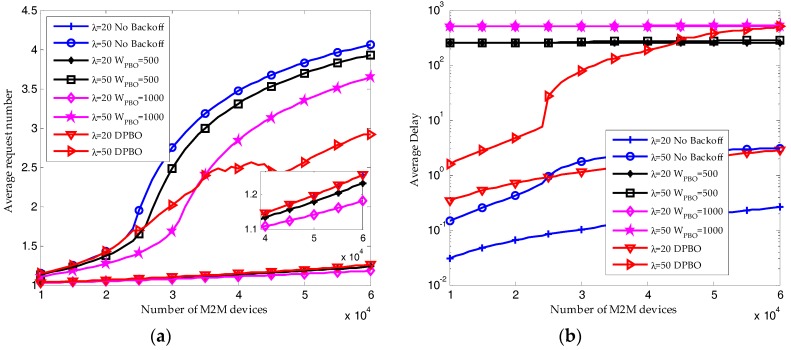
Performance results of HSTMAC protocol with dynamic pre-backoff scheme (Q = 10, W_BO_ = 20): (**a**) Average request number of M2M traffic; (**b**) Average delay of M2M traffic (granularity in cycles).

When the arrival rate of H2H packets is *λ* = 50, the system quickly becomes congested due to the growth in the number of M2M devices. Shown in Figure 12a and Figure 13a, the performance results without backoff scheme are the worst. On the other hand, though the average success probability is decreasing with the number of M2M devices, the results in DPBO algorithm is better than that in scenarios with UPBO algorithm. In a scenario with high system load, as shown in Figure 12b and Figure 13a, the resource utilization ratio in DPBO algorithm is higher while the average request number is lower than that in UPBO algorithm. Moreover, as shown in Figure 13b, the average delay in DPBO algorithm is lower or in the same order with that in UPBO algorithm.

As a conclusion, we can derive that pre-backoff scheme can improve the success probability at cost of lowering the resource utilization ratio and increasing the delay. However, the proposed DPBO algorithm can improve the system performance at a much lower cost when compared to the UPBO algorithm.

## 7. Conclusions

In order to jointly optimize the random access procedure and the following data transmission procedure, we propose a hybrid S-ALOHA/TDMA MAC protocol to address the radio access network congestion problem caused by massive M2M devices. We prove that there exists a packet size threshold $\bar{\delta }$ when considered the signaling overhead in the random access procedure. The S-ALOHA protocol has superiority in small data transmission when $\delta <\bar{\delta }$ and the TDMA protocol has superiority in large data transmission when $\delta >\bar{\delta }$. Then a more rigorous system equilibrium analysis is derived and the results demonstrate that the system performance can be improved dramatically by allocating more resource blocks when it is in a saturation state. However, the improvement becomes negligible when the system is in a non-saturation state. Therefore, the equilibrium analysis could provide the operator with more insights on QoS provision. At last, for the load balance of beta distributed M2M traffic, we design a dynamic pre-backoff scheme. The performance results show that the proposed HSTMAC protocol is superior to both the pure S-ALOHA protocol and pure TDMA protocol in terms of average success probability and resource utilization ratio, especially when the system load is high. The results also demonstrate that the proposed DPBO algorithm can improve the access success probability and resource utilization ratio at a much lower cost in delay when compared to the uniform pre-backoff algorithm.

## Figures and Tables

**Table 1:** Performance Evaluation Parameters for Proposed HSTMAC Protocol.

Parameter	Settings
Cell bandwidth	10 MHz (50 RBs in frequency domain)
Cycle interval τ	5 ms
Reserved RBs for uplink transmission L	200
Transmission limit Q	5, 10
H2H traffic model	Poisson distribution
M2M traffic model	Beta distribution
Signaling overhead parameter for H2H traffic γ	2
Packet size of H2H traffic $\delta_{1}$	2
Packet size of M2M traffic $\delta_{2}$	0.5
Number of M2M devices $N^{M2M}$	10,000–60,000
Activation time of M2M devices T	10 s
Beta function parameters α,β	3, 4
Backoff window size for M2M traffic $W_{BO}$	20
DPBO time control parameter for M2M traffic η	10

**Table 2:** Success Probability at the Saturation Threshold under Different Transmission Limit.

Q	1	2	3	4	5	6	7	8	9	10
$P_{S}$	0.3679	0.6004	0.7474	0.8403	0.8991	0.9362	0.9597	0.9745	0.9839	0.9898
